# Design rules for scalability in spin-orbit electronics

**DOI:** 10.1038/s41598-019-49831-5

**Published:** 2019-09-24

**Authors:** Mohammad Kazemi, Mark F. Bocko

**Affiliations:** 1 Department of Electrical and Computer Engineering, University of Rochester, Rochester, New York 14627 USA; 2 Department of Physics and Astronomy, University of Rochester, Rochester, New York 14627 USA

**Keywords:** Electrical and electronic engineering, Electrical and electronic engineering, Magnetic devices, Magnetic devices, Magnetic devices

## Abstract

Spin-orbit electronics (spin-orbitronics) has been widely discussed for enabling nonvolatile devices that store and process information with low power consumption. The potential of spin-orbitronics for memory and logic applications has been demonstrated by perpendicular anisotropy magnetic devices comprised of heavy-metal/ferromagnet or topological-insulator/ferromagnet bilayers, where the heavy metal or topological insulator provides an efficient source of spin current for manipulating information encoded in the bistable magnetization state of the ferromagnet. However, to reliably switch at room temperature, spin-orbit devices should be large to reduce thermal fluctuations, thereby compromising scalability, which in turn drastically increases power dissipation and degrades performance. Here, we show that the scalability is not a fundamental limitation in spin-orbitronics, and by investigating the interactions between the geometry of the ferromagnetic layer and components of the spin-orbit torque, we derive design rules that lead to deeply scalable spin-orbit devices. Furthermore, employing experimentally verified models, we propose deeply scaled spin-orbit devices exhibiting high-speed deterministic switching at room temperature. The proposed design principles are essential for design and implementation of very-large-scale-integration (VLSI) systems that provide high performance operation with low power consumption.

## Introduction

Scaling of complementary-metal-oxide-semiconductor (CMOS) transistors has yielded steady improvement in performance and reduction in power dissipation of computing systems over the past five decades^1,2^. As CMOS transistors approach the intrinsic limits of scalability, higher performance requires drastically more power dissipation and circuit area. The advent of spin-orbit magnetic heterostructures^3^, such as heavy-metal/ferromagnet^4–10^ and topological-insulator/ferromagnet^11–15^ thin films, renders magnetic devices promising for the implementation of power efficient memory, logic, and machine learning systems. A charge current flowing into the heavy metal or topological insulator produces a non-equilibrium spin accumulation at the surface. By coupling a ferromagnet film to the accumulated spin, the flow of spin angular momentum from the spin current produces a torque on the magnetization, which is known as the spin-orbit torque. Recent studies have projected that the spin-orbit torque might provide an energy efficient mechanism (≤10^−14^ Joule) for high speed switching (≤10^−9^ Second) of the magnetization in perpendicular-anisotropy magnetic devices^6,11^.

The energy efficiency and performance of computing systems are determined primarily by the integration density of the underlying technology^1,2^. Hence, scalability is essential for any technology to support computing applications. While the efficiency of various material systems for producing spin-orbit torques has been extensively investigated employing micron sized magnetic heterostructures^4–13^, scalability of spin-orbit magnetic devices has remained an outstanding challenge. The two key performance metrics that must be preserved while scaling the device dimensions to deep sub-micron sizes are data retention over a long period of time (nonvolatility) and deterministic switching operation (reliability) at room temperature.

To assure nonvolatility, the stable magnetization states should be separated by a sufficiently large energy barrier (≥40 *k*_*B*_*T*). Here *k*_*B*_ is Boltzmann’s constant and *T* is temperature in Kelvin. The energy barrier is proportional to the magnetic anisotropy energy density and volume of the ferromagnetic layer. As the volume of the ferromagnet is reduced by scaling the device dimensions, to ensure nonvolatility, the antisymmetric exchange interactions^16–18^ should be suppressed and the anisotropy energy density should be increased. The antisymmetric exchange interactions induce canted instead of aligned spin states, which renders the device prone to thermal fluctuations by inducing sub-volume excitation^19^ of the ferromagnetic layer. Devices with perpendicular magnetic anisotropy (PMA) are strongly preferred over those with in-plane magnetic anisotropy (IMA) for nonvolatile operation. In contrast to the IMA originating from the device shape anisotropy, the PMA can originate from the interface between the magnetic and non-magnetic films within the device^4,5,20^. Thus, the PMA is not limited by the planar geometry of the device and may extend to relatively large values required for nonvolatility at smaller dimensions.

While rapid advances in fabrication of nonvolatile spin-orbit devices with scaled dimensions can be expected, it is of significant technological relevance to derive design rules that allow such devices to exhibit reliability, i.e., deterministic switching at room temperature. In this paper, we present design rules for achieving deeply scalable spin-orbit magnetic devices that exhibit deterministic switching operation at room temperature (≥300 *K*). We further show that the design rules enable high speed switching operation with low power consumption, suggesting the spin-orbitronics as a promising candidate for post-CMOS systems delivering high performance operation with low power consumption.

## Geometry as a Degree of Freedom for Scalability in Spin-Orbitronics

**Figure 1:**
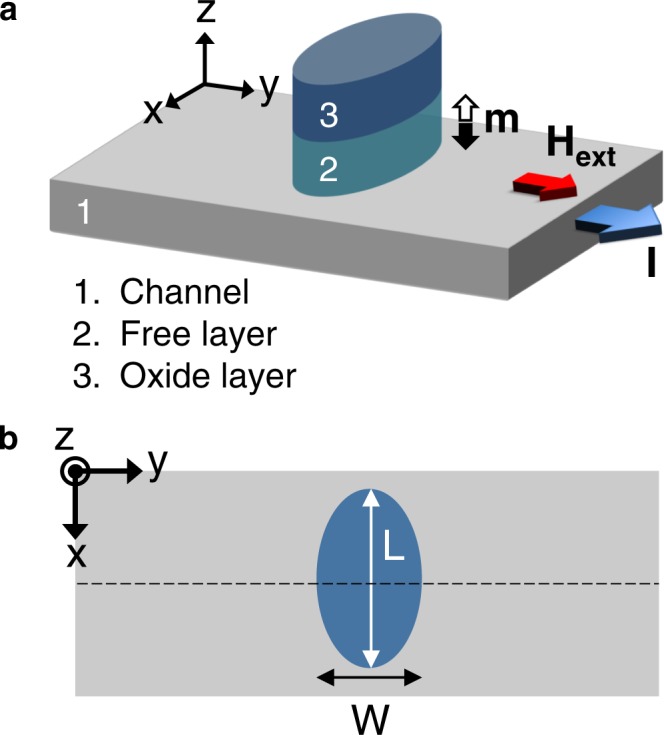
Device structure. (**a**) Layer architecture of the fundamental building block of magnetic devices that employ spin-orbit torque to manipulate a bit encoded in the bistable magnetization state of a ferromagnetic layer. The channel is a material with strong spin-orbit interaction, and the free layer is made of a ferromagnetic material. (**b**) Top view of the device. To study the effect of geometry on the device behavior, an elliptical free layer, instead of a conventional circular layer, is considered. The nominal device has the following parameters: channel width 40 *nm*, channel thickness 5 *nm*, free layer area equivalent to a circle with a radius of 12 *nm*, free layer thickness 1.2 *nm*, perpendicular anisotropy field ${H}_{PA}=4,000\,Oe$, saturation magnetization ${M}_{s}=1,550\,emu/c{m}^{3}$, and damping factor $\alpha \in [0.02,0.1]$.

In this section, we introduce and study the planar geometry of the ferromagnetic layer as a degree of freedom for achieving deeply scalable spin-orbit devices. The schematic of the studied spin-orbit magnetic device is illustrated in Fig. 1. This heterostructure represents the fundamental building block of the magnetic devices that employ spin-orbit torque to manipulate information encoded in the bistable magnetization state of a ferromagnet. The device comprises a ferromagnetic layer, referred to as the free layer, which is sandwiched between an oxide layer and a thin film with strong spin-orbit interaction, referred to as the channel. The magnetization of the free layer can be switched between two stable states along the *z* axis (+*z* and −*z*) via the spin-orbit torque produced by injecting a current pulse into the channel. To study the effect of geometry on device operation at scaled dimensions, the ferromagnetic layer is considered to be elliptical with a width and length of *W* and *L*, respectively. The magnetic dynamics of the device in response to the current-induced spin-orbit torque is investigated by numerical integration of the stochastic Landau-Lifschitz-Gilbert-Slonczewski (LLGS) equation^21^ and via experimentally verified models of spin-orbit heterostructures^22^.

The magnetization of the free layer is determined by the effect of the torques and fields at room temperature. The spin-orbit torque (**T**_*SOT*_) comprises two primary components, a damping-like torque (**T**_*DL*_) and a field-like torque (**T**_*FL*_). Denoting the unit vector along the magnetization by **m** and along the spin polarization by ***σ***, **T**_*SOT*_ is represented as

1 $$\begin{array}{rcl}{{\bf{T}}}_{SOT} & = & {{\bf{T}}}_{DL}+{{\bf{T}}}_{FL}\\  & = & \frac{\hslash }{2e}\frac{{{\bf{J}}}_{e}}{{M}_{s}{t}_{FL}}\{{\zeta }_{DL}{\bf{m}}\times ({\boldsymbol{\sigma }}\times {\bf{m}})+{\zeta }_{FL}{\boldsymbol{\sigma }}\times {\bf{m}}\},\end{array}$$

where ${\zeta }_{DL}$ and ${\zeta }_{FL}$ denote the efficiency of the channel current in producing the damping-like and field-like torque, respectively. Here, $\hslash$, *e*, **J**_*e*_, *M*_*s*_, and *t*_*FL*_ denote the reduced Planck’s constant, electron charge, channel current density, saturation magnetization, and free layer thickness, respectively. We choose the thickness of the free layer to be 1.2 *nm* and the area of the free layer to be equivalent to a circle with a radius of 12 *nm*. Accordingly, to ensure nonvolatility (Energy barrier = 40 *k*_*B*_*T*), the saturation magnetization and the PMA field are set to, respectively, ${M}_{s}=1,550\,emu/c{m}^{3}$ and ${H}_{PA}=4,000\,Oe$, which are achievable in a wide range of spin-orbit magnetic heterostructures. The interfacial perpendicular anisotropy *K*_*i*_ is related to *H*_*PA*_ via $\frac{{K}_{i}}{{t}_{FL}}\simeq \frac{1}{2}{M}_{s}{H}_{PA}+2\pi {M}_{s}^{2}{N}_{z}$, where *t*_*FL*_ is the thickness of the free layer, and *N*_*z*_ denotes the demagnetizing factor along the *z* axis. The *K*_*i*_ corresponding to ${H}_{PA}=4,000\,Oe$, ${M}_{s}=1,550\,emu/c{m}^{3}$, and ${t}_{FL}=1.2\,nm$ is about $1.9\,erg/c{m}^{2}$ (*N*_*z*_ = 0.86) which lies in the range of practically achievable values^20^.

**Figure 2:**
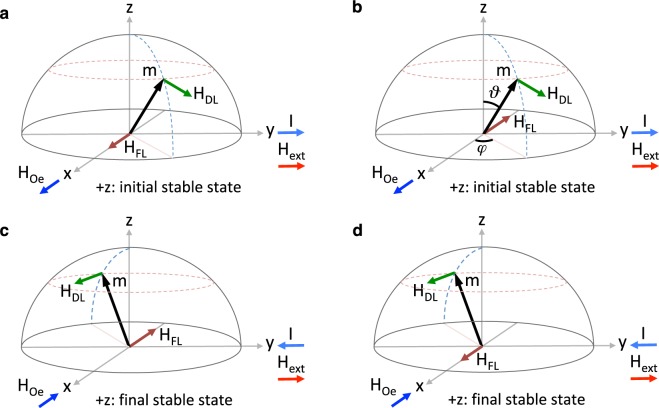
Current induced magnetic fields. The current induced Oersted field is denoted as **H**_*Oe*_, and the current induced effective fields corresponding to the field-like and damping-like spin-orbit torques are denoted, respectively, as **H**_*FL*_ and **H**_*DL*_. **H**_*DL*_ lies within the *y*–*z* plane and has no component along the *x* axis (the spin-polarization axis). **H**_*DL*_ always destabilizes the magnetization, i.e., (**a**,**b**) moves the magnetization away from its initial stable state, and (**c**,**d**) keeps the magnetization at an equilibrium state away from the final stable state (at the equilibrium, the resultant of all fields experienced by the magnetization is zero). Alternatively, **H**_*FL*_ lies along the *x* axis and has no component in the *y*–*z* plane. Depending on the sign of ${\zeta }_{FL}$, which characterizes the capability of the material system to produce the field-like torque, **H**_*FL*_ may add to **H**_*Oe*_ as shown in **a** and **c**, or subtract from **H**_*Oe*_ as illustrated in (**b** and **d**).

Detailed geometric and magnetic parameters of the device are provided in the caption of Fig. 1. **T**_*DL*_ and **T**_*FL*_ can be represented as being induced by two spin-orbit effective fields ${{\bf{H}}}_{DL}=H{\zeta }_{DL}{\bf{m}}\times {\boldsymbol{\sigma }}$ and ${{\bf{H}}}_{FL}=H{\zeta }_{FL}{\boldsymbol{\sigma }}$, where $H=\frac{\hslash }{2e}\frac{{{\bf{J}}}_{e}}{{M}_{s}{t}_{FL}}$. The channel current also produces an Oersted field along the *x* axis, denoted by **H**_*Oe*_ in Fig. 2, which is collinear to **H**_*FL*_. Depending on the sign of ${\zeta }_{FL}$, **H**_*Oe*_ may add to or subtract from **H**_*FL*_. To characterize the effect of **H**_*Oe*_ on device operation, we define ${\zeta }_{FL}^{eff}$ such that ${{\bf{H}}}_{Oe}+{{\bf{H}}}_{FL}=H{\zeta }_{FL}^{eff}{\boldsymbol{\sigma }}$. Furthermore, to enable the polarity of the channel current (+*y* or −*y*) to favor a unique stable state of the magnetization (+*z* or −*z*), an external magnetic field (**H**_*ext*_) is applied along the channel to break the symmetry^4,5^. **H**_*ext*_ is usually produced by integrating a nanomagnet with the device, and is taken to be small (≤0.1 *H*_*PA*_) to avoid compromising device nonvolatility via reduction of the energy barrier between the stable magnetization states.

Sufficiently large PMA is essential for nonvolatile retention of a stable magnetization state at room temperature. However, since the switching process is influenced by the entire magnetic energy landscape, even a large PMA may not yield reliable switching in deeply scaled devices. We investigate shaping the magnetic energy landscape for achieving reliable switching at scaled dimensions. To analyze the effect of the magnetic energy landscape on switching reliability, we characterize the energy saddle points and investigate how the position of the saddle points with respect to the spin polarization axis (*x* axis) influences the switching reliability. ${{\bf{H}}}_{FL}+{{\bf{H}}}_{Oe}$ is first assumed to be zero (i.e., ${\zeta }_{FL}^{eff}=0$). The analysis is then continued by considering the effect of ${{\bf{H}}}_{FL}+{{\bf{H}}}_{Oe}$ on device operation. In the analysis, we assume that ${\zeta }_{DL} > 0$. Due to symmetry, all results are also valid for devices with ${\zeta }_{DL} < 0$.

Before going through the analysis, we define three distinguishable states which the magnetization takes during a switching event: the initial stable state, the final stable state, and the equilibrium state. The initial stable state refers the stable magnetization state before injecting a current pulse into the channel, the final stable state refers the stable magnetization state after current pulse injection, and the equilibrium state refers to the state where the resultant of all fields experienced by the magnetization is zero.

**Figure 3:**
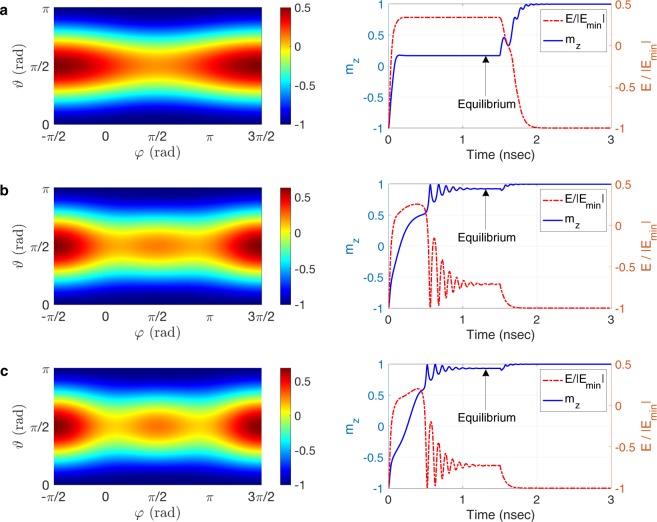
The effect of geometry on the magnetic energy landscape and switching dynamics. Normalized magnetic energy landscape $(\tfrac{E}{|{E}_{{\min }}|})$, switching trajectory, and normalized switching energy path for a device with the free layer length and width of (**a**) $L=24\,nm$ and $W=24\,nm$, (**b**) $L=32\,nm$ and $W=18\,nm$, and (**c**) $L=36\,nm$ and $W=16\,nm$. By increasing the shape anisotropy (free layer aspect ratio), the energy saddle points move toward the spin polarization axis (*x* axis), and the equilibrium is reached at a significantly lower energy state. ${\vartheta }$ and $\phi$ denote the angle which the magnetization makes, respectively, with the *z* axis and with the *x* axis at each time instant.

**Figure 4:**
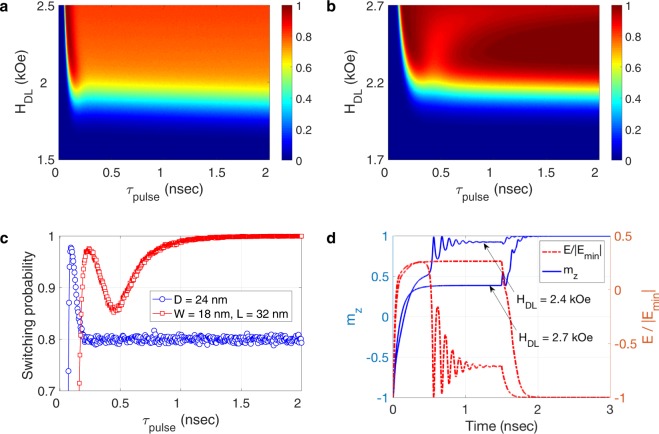
Switching probability diagrams (SPDs) and switching trajectories exhibiting the geometry effect on device reliability. The SPD corresponding to the switching events for the device with (**a**) the circular free layer with a diameter of $D=24\,nm$, and (**b**) the elliptical free layer with a width of $W=18\,nm$ and a length of $L=32\,nm$. While the switching probability does not exceed 0.8 over a wide range of pulse parameters for the circular device, a high switching probability of larger than 0.9995 is achievable for the device with shaped magnetic energy landscape. (**c**) Switching probabilities corresponding to events caused by a current pulse producing ${H}_{DL}=2.4\,kOe$. (**d**) Switching trajectories for the device with shaped magnetic energy landscape, induced by two pulses with the same duration (1.5 *nsec*) but different amplitudes (producing ${H}_{DL}=2.4\,kOe$ and 2.7 *kOe*). The calculations were repeated 10,000 times for each pulse duration and amplitude.

The magnetic energy landscape for three devices with the same volume but different aspect ratios of the free layer are illustrated in Fig. 3. For the device with free layer aspect ratio of $\eta =1$ (circular cross section), the saddle point lies along the current flow (*y* axis), as illustrated in Fig. 3a. Since the spin-orbit torque tends to align the magnetization with the spin polarization direction (*x* axis), the magnetization reaches the equilibrium at an energetically excited state which is highly susceptible to thermal fluctuations, leading to stochastic switching. As shown in Fig. 4a,c, the switching probability for the device does not exceed 0.8 over a wide range of the current pulse duration (${\tau }_{pulse}$) and amplitude (*J*_*e*_; ${H}_{DL}=\tfrac{\hslash }{2e}\tfrac{{\zeta }_{DL}}{{M}_{s}{t}_{FL}}{J}_{e}$), which is too low to be considered reliable. Although a PMA field of ${H}_{PA}=4,000\,Oe$ is sufficient to retain a stable magnetization state for a long period of time at room temperature in this device, a prohibitively larger PMA field may be required to overcome thermal fluctuations and stabilize the magnetization from the equilibrium to the final stable state. Switching power dissipation is quadratically proportional to the PMA field^23^. Hence, relying on a prohibitively large PMA for reliable switching is not a viable solution as a large PMA drastically increases switching power dissipation, thus compromising scalability.

We propose employing the planar geometry of the free layer as a degree of freedom for shaping the magnetic energy landscape to achieve reliable switching, without the need for increasing the PMA field beyond the level required for nonvolatility. By introducing in-plane magnetic shape anisotropy into the free layer via increasing the in-plane aspect ratio of the free layer, the saddle points of the magnetic energy landscape move toward the spin polarization axis (*x* axis). The position of the saddle points in the polar coordinate system is given by $({{\vartheta }}_{s},{\phi }_{s})=(\frac{\pi }{2},{\sin }^{-1}(\frac{{H}_{ext}}{{H}_{IA}}))$, where *H*_*IA*_ denotes the in-plane anisotropy field produced by the shape anisotropy. Hence, in contrast to the circular geometry, the switching trajectory will pass through the saddle region of the magnetic energy landscape for a wide range of current pulse parameters, reaching equilibrium in a low-energy state within the duration of the current pulse, as shown in Fig. 3b,c. Hence, after current pulse injection, even a small PMA field is sufficient to absorb the magnetization effectively from the equilibrium state toward the final stable state, leading to robust switching operation in the presence of thermal fluctuations, as illustrated in Fig. 4b. For the device with the same magnetic parameters and volume of the free layer as described above, a free layer aspect ratio of $\eta =\frac{32}{18}$ is sufficient to achieve a switching probability larger than 0.9995 over a wide rage of the amplitude and duration of the channel current (Switching Error Rate (SER) < 5 × 10^−4^). Accordingly, nonvolatility and reliability can be achieved by tuning two independent degrees of freedom, the perpendicular magnetic anisotropy and the in-plane magnetic anisotropy, respectively. Consequently, the presence of in-plane magnetic anisotropy is essential for reliable operation at scaled dimensions.

In striking contrast to the two terminal magnetic devices operating via spin-transfer torque (STT)^24^, larger current densities may not necessarily lead to higher switching speeds in scaled devices operating on the basis of spin-orbit torque. The fields with *z*-component operating on magnetization are **H**_*DL*_ and **H**_*PA*_; at equilibrium, ${{\bf{H}}}_{DL}\cdot \hat{z}+{{\bf{H}}}_{PA}\cdot \hat{z}=0$. Hence, as shown in Fig. 2, while driving the magnetization out of the initial stable state, the damping-like torque keeps the magnetization away from the final stable state once the magnetization reaches equilibrium (i.e., works opposite to the stabilizing torque exerted by **H**_*PA*_ on the magnetization). By increasing the channel current density, the magnetization arrives more quickly at the equilibrium state. Nevertheless, the net destabilizing effect of the spin-orbit torque on the equilibrium state increases as well, leading to an equilibrium state that is energetically farther away from the final stable state, as shown in Fig. 4d. Thus, the magnetization takes longer to stabilize to the final stable state after current pulse injection and the switching process becomes prone to thermal fluctuations, which dramatically increases the switching error rate (more than three orders of magnitude).

## Deeply Scalable Spin-Orbit Devices with High Speed Switching at Room Temperature

Having characterized the geometry as a degree of freedom for achieving deeply scaled spin-orbit devices, we introduce a design principle for achieving sub-nanosecond deterministic switching at deeply scaled dimensions. Spin-orbit heterostructures exhibit a wide range of damping-like and field-like torques. Furthermore, since the free layer is fabricated immediately on the channel, the effect of **H**_*Oe*_ on the dynamics of the free layer magnetization is significant. So far, assuming that ${{\bf{H}}}_{FL}+{{\bf{H}}}_{Oe}=0$ (equivalently, ${\zeta }_{DL}^{eff}=0$), we have not considered the effect of the field-like torque and **H**_*Oe*_ on device operation. In the following, we show that the field-like torque and **H**_*Oe*_ may dramatically affect both the switching speed and reliability at scaled dimensions.

**Figure 5:**
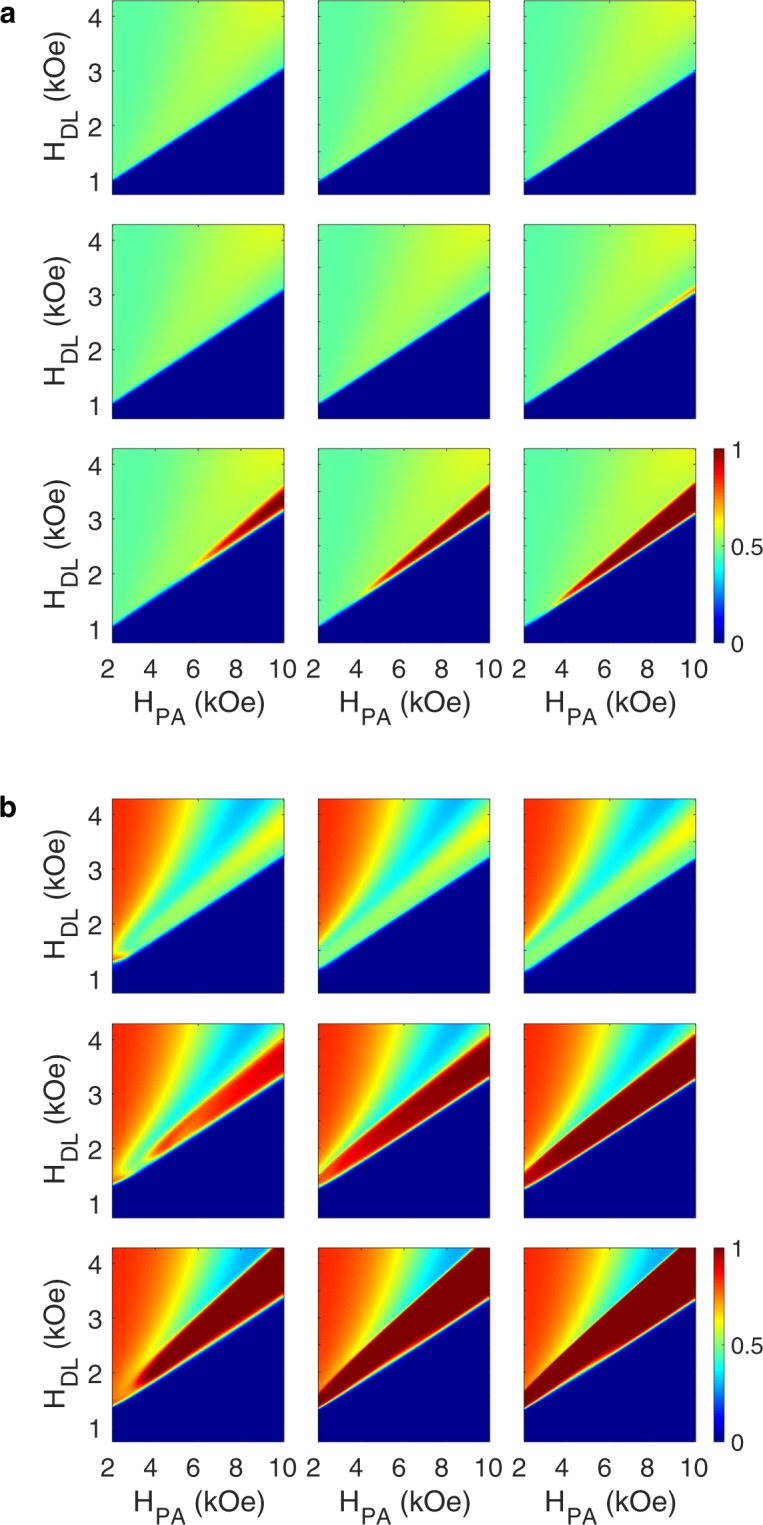
Switching probability diagrams (SPDs) characterizing the role of the effective torque ratio $\tfrac{{\zeta }_{FL}^{eff}}{{\zeta }_{DL}}$ on switching reliability and speed. The SPDs corresponding to the switching events for the device with (**a**) the circular free layer with a diameter of 24 *nm*, and (**b**) the elliptical free layer with a width of 18 *nm* and a length of 32 *nm*. SPDs at each row correspond to switching events produced by a current pulse with a duration of, from left to right, 0.5 ns, 1 ns, and 2 ns. SPDs at each column correspond to switching events in a device with an effective torque ratio $\tfrac{{\zeta }_{FL}^{eff}}{{\zeta }_{DL}}$ of, from top to bottom, −0.05, 0, and 0.05. By increasing $\tfrac{{\zeta }_{FL}^{eff}}{{\zeta }_{DL}}$ from −0.05 to 0.05, high speed deterministic switching is enabled in deeply scaled devices with shaped magnetic energy landscape. Positive $\tfrac{{\zeta }_{FL}^{eff}}{{\zeta }_{DL}}$ also slightly enhances switching success rate for the circular device, but only at very large PMA fields which are not practical. The calculations were repeated 10,000 times for each pixel.

**H**_*DL*_ lies in the *y*–*z* plane, thus the damping-like torque induces a rotational motion of the magnetization between the stable magnetization states (+*z* and −*z*). **H**_*FL*_ lies along the *x* axis, so the field-like torque produces a precessional motion of the magnetization around the *x* axis. Depending on whether the effective torque ratio, characterized by $\frac{{\zeta }_{FL}^{eff}}{{\zeta }_{DL}}$, is negative or positive, the *z*-component of the effective field-like torque (${{\bf{T}}}_{FL}^{z}={{\bf{T}}}_{FL}\cdot \hat{z}$) may, respectively, add to or subtract from the *z*-component of the damping-like torque (${{\bf{T}}}_{DL}^{z}={{\bf{T}}}_{DL}\cdot \hat{z}$) at equilibrium. Accordingly, for a negative $\frac{{\zeta }_{FL}^{eff}}{{\zeta }_{DL}}$, the stabilizing torque produced by **H**_*PA*_ should overcome a larger destabilizing spin-orbit torque. The equilibrium state then lies at a relatively high-energy state close to the *x*–*y* plane, which is prone to thermal fluctuations and hence leads to stochastic switching. Alternatively, for a positive $\frac{{\zeta }_{FL}^{eff}}{{\zeta }_{DL}}$, ${{\bf{T}}}_{FL}^{z}$ largely cancels out ${{\bf{T}}}_{DL}^{z}$ at equilibrium, allowing the magnetization to relax to a low-energy equilibrium state close to the final stable state. The magnetization then quickly stabilizes toward the final stable state after current pulse injection, even in heterostructures without a large damping factor ($\alpha  < 0.03$). As illustrated in Fig. 5, the switching can therefore be accomplished rapidly (<1 *ns*), and the process is highly robust to thermal fluctuations, leading to deterministic switching (SER < 10^−4^), which is essential for memory and logic applications.

Accordingly, an effective approach to substantially accelerate the switching operation while maintaining the reliability is to cancel the effect of the damping-like torque at the equilibrium state to a greater degree. This can be achieved by increasing the positive torque ratio $\frac{{\zeta }_{FL}^{eff}}{{\zeta }_{DL}}$. The magnetization then not only moves more quickly toward the equilibrium state, but also reaches an equilibrium state closer to the final stable state, which shortens the stabilization time after current pulse injection. As illustrated in Fig. 5, $\frac{{\zeta }_{FL}^{eff}}{{\zeta }_{DL}}=0.05$ enables deterministic switching with pulses as short as 500 ps and an amplitude in the range of the device critical current, without the need for a PMA field larger than that required to ensure nonvolatility. Surprisingly, subnanosecond switching time is enabled even at PMA fields smaller than those needed for nonvolatility, which is critical for applications requiring short retention time, such as high level cache memories and a wide range of logic systems. Hence, since power dissipation is reduced significantly (quadratically) by decreasing the PMA field, nonvolatility can be traded off for power dissipation, without compromising a device reliability. The derived design rules, therefore, may enable spin-orbitronics to serve as a *universal memory technology*, with high speed operation, low power dissipation, and high integration density, which has been a long-standing quest^25^.

## Discussion

Realizing heterostructures with positive torque ratio $\frac{{\zeta }_{FL}^{eff}}{{\zeta }_{DL}}$ paves the way toward deeply scalable spin-orbit devices with high speed deterministic switching. In heterostructures with positive ${\zeta }_{DL}$, such as those with a channel composed of Pt (refs^4^ and^5^) or Bi_2_Se_3_ (refs^11–13^), the Oersted field produced by the channel current (**H**_*Oe*_) is always antiparallel to **H**_*FL*_. Hence, although in such heterostructures the torque ratio $\frac{{\zeta }_{FL}}{{\zeta }_{DL}}$ is negative, a positive *effective* torque ratio $\frac{{\zeta }_{FL}^{eff}}{{\zeta }_{DL}}$ is achievable via suppressing **H**_*FL*_ (ref.^5^). As discussed above, even a small positive $\frac{{\zeta }_{FL}^{eff}}{{\zeta }_{DL}}$ (~0.05) is sufficient for achieving high-speed reliable switching at deeply scaled spin-orbit devices.

**Figure 6:**
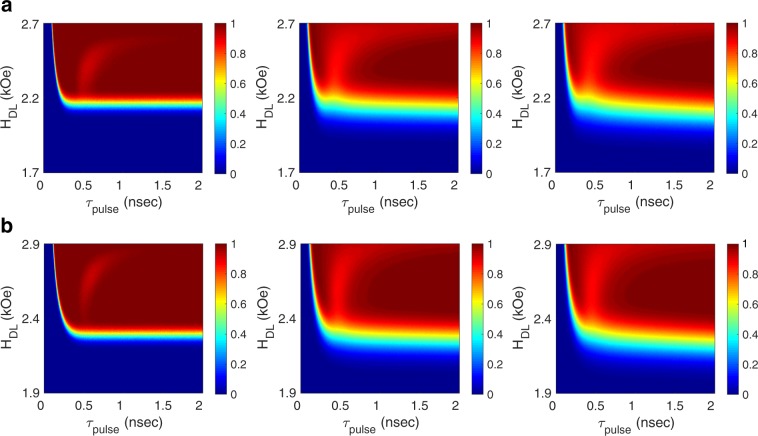
The effect of free layer aspect ratio on switching reliability as a function of temperature. The SPDs corresponding to the switching events for a device with (**a**) the aspect ratio of $\eta =\frac{32}{18}$, and (**b**) the aspect ratio of $\eta =\frac{36}{16}$. SPDs at each row correspond to switching events at, from left to right, $T=50\,K$, $T=300\,K$, and $T=398\,K$. All parameters but the free layer aspect ratio are the same for both devices. The device with a larger aspect ratio achieves deterministic switching over a relatively wider pulse parameters at higher temperatures. However, while having significantly different aspect ratios, both devices exhibit deterministic switching over a wide range of current pulse parameters, implying that the precise control of the aspect ratio is not necessary.

As temperature increases, thermal fluctuations become stronger. Hence, the role of in-plane magnetic shape anisotropy in achieving deterministic switching increases at higher temperatures. Figure 6 illustrates the switching probability diagrams for two devices at three different temperatures, namely, the cryogenic temperature $T=50\,K$, the room temperature $T=300\,K$, and the on-chip temperature $T=398\,K$. All parameters but the aspect ratio of the free layer are the same for both devices. Particularly, for both devices ${H}_{PA}=4,000\,Oe$ (${K}_{i}=1.9\,erg/c{m}^{2}$), ${M}_{s}=1,550\,emu/c{m}^{3}$, ${t}_{FL}=1.2\,nm$, and the area of the free layer is equivalent to the area of a circle with a radius of 12 *nm*. As illustrated in Fig. 6, at cryogenic temperature both devices achieve deterministic switching for a wide range of current pulse parameters. At room temperature and on-chip temperature, however, the device with the larger aspect ratio $\eta =\frac{36}{16}$ operates reliably over a relatively wider range of current pulse parameters.

Although the presence of an in-plane magnetic shape anisotropy is essential for deterministic switching in scaled devices, the precise control of the free layer aspect ratio is not necessary. Particularly, deterministic switching can be achieved for a wide range of free layer aspect ratios, thus relaxing the fabrication process of scaled spin-orbit devices via eliminating the need for precise control of the aspect ratio. As illustrated in Fig. 6, aspect ratio of the free layer may significantly vary, yet the device exhibits reliable switching at scaled dimensions over a wide range of temperatures, verifying that for scalability the device aspect ratio is not required to be precisely controlled. Also, as illustrated in Fig. 6, by increasing the free layer aspect ratio largely from $\eta =\frac{32}{18}$ to $\eta =\frac{36}{16}$, the switching current increases by about 10%. Accordingly, adding a suitable margin (about 10%) to the channel current with respect to the minimum switching current ensures deterministic switching in the worst case scenario (corresponding to $\eta =\tfrac{36}{16}$).

## Conclusions

In conclusion, we show that scalability is not a fundamental limitation in spin-orbitronics and provide design rules for achieving deeply scalable spin-orbit devices with high speed switching at room temperature. We introduce and comprehensively study the geometry as a degree of freedom for scalability in spin-orbitronics. We show that the interaction of the spin-orbit torque components with the magnetic energy landscape, governed by the ferromagnetic layer geometry, is crucial to switching reliability, speed, and power dissipation in spin-orbit devices with deeply scaled dimensions. The design principles introduced and investigated in this paper pave the way toward the application of spin-orbitronics in very-large-scale-integration (VLSI) systems, such as memories, processors, and neural networks, with the capability to deliver high performance operation under the limitations imposed by low power consumption.

## Methods

### Thermal fluctuations model

A nonzero temperature introduces thermal fluctuations to the magnetization, which is modeled by the Langevin random field ${{\bf{H}}}_{L}=({H}_{L,x},{H}_{L,y},{H}_{L,z})$. Each component of **H**_*L*_ follows a zero-mean Gaussian random process whose standard deviation is a function of temperature^26,27^,

2 $$\delta =\sqrt{\frac{2\alpha {k}_{B}T}{\gamma {M}_{s}{v}_{F}\Delta t}}.$$

Here *α*, *k*_*B*_, *T*, *M*_*s*_, and *v*_*F*_ denote, respectively, the damping factor, Boltzmann constant, temperature, saturation magnetization, and volume of the free layer. Δ*t* is the duration of the constant effective thermal fluctuation field. The standard deviation of the thermal fluctuations is inversely proportional to the volume of the free layer. Hence, by scaling the free layer dimensions, **H**_*L*_ becomes stronger, rendering the magnetization dynamics more susceptible to thermal fluctuations.

### Calculation of the magnetic energy landscape

The magnetic energy landscape is governed by three components, (1) the uniaxial magnetic anisotropy, (2) the demagnetizing field, and (3) the external magnetic field. Accordingly,

3 $$\begin{array}{rcl}E({\bf{m}}) & = & {E}_{PA}+{E}_{DM}+{E}_{H}\end{array}$$

4 $$\begin{array}{rcl} & = & -{K}_{u}{({\bf{m}}.z)}^{2}+2\pi {{M}_{s}}^{2}({N}_{x}{m}_{x}^{2}+{N}_{y}{m}_{y}^{2}+{N}_{z}{m}_{z}^{2})-{M}_{s}{\bf{m}}.{{\bf{H}}}_{{\bf{e}}{\bf{x}}{\bf{t}}},\end{array}$$

where *E*_*PA*_, *E*_*DM*_, and *E*_*H*_ denote the energy components due to, respectively, the perpendicular magnetic anisotropy, demagnetizing field, and the external magnetic field. Here, *K*_*u*_ is the perpendicular magnetic anisotropy, $${\bf{N}}=({N}_{x},{N}_{y},{N}_{z})$$ is the demagnetizing tensor, where $${N}_{x}+{N}_{y}+{N}_{z}=1$$, and $${\bf{m}}=({m}_{x},{m}_{y},{m}_{z})=(\sin ({\vartheta })\,\cos (\phi ),$$$$\sin ({\vartheta })\,\sin (\phi ),\,\cos ({\vartheta }))$$ is a unit vector along the magnetization.
